# YLR419W is the homolog of the mammalian translation initiation factor *DHX29*

**DOI:** 10.17912/micropub.biology.001112

**Published:** 2024-02-22

**Authors:** Micheline Fromont-Racine, Varun Khanna, Alain Jacquier, Gwenael Badis

**Affiliations:** 1 Cytoplasmic mRNA surveillance in yeast, Institut Pasteur, Paris, Île-de-France, France; 2 UMR3525, French National Centre for Scientific Research, Paris, Île-de-France, France; 3 Institut Pasteur, Paris, Île-de-France, France

## Abstract

27 years after the yeast genome sequencing, the function of many ORFs remain unknown. Despite the evolutionary distance between human and yeast, homology with the conserved DEAH/DExH-box helicase domains allowed us to list DHX29, DHX36 and DHX57 as three putative homologs of the yeast Ylr419wp. Functional studies first linked the Ylr419w protein to the translating ribosome and cross-linking and analysis of cDNA (CRAC) experiments determined the precise region of Ylr419wp in contact with the ribosome. It corresponds to the loop of the h16 helix in the 18S rRNA designing the translation initiation factor DHX29, as the functional homolog of Ylr419wp.

**Figure 1.**
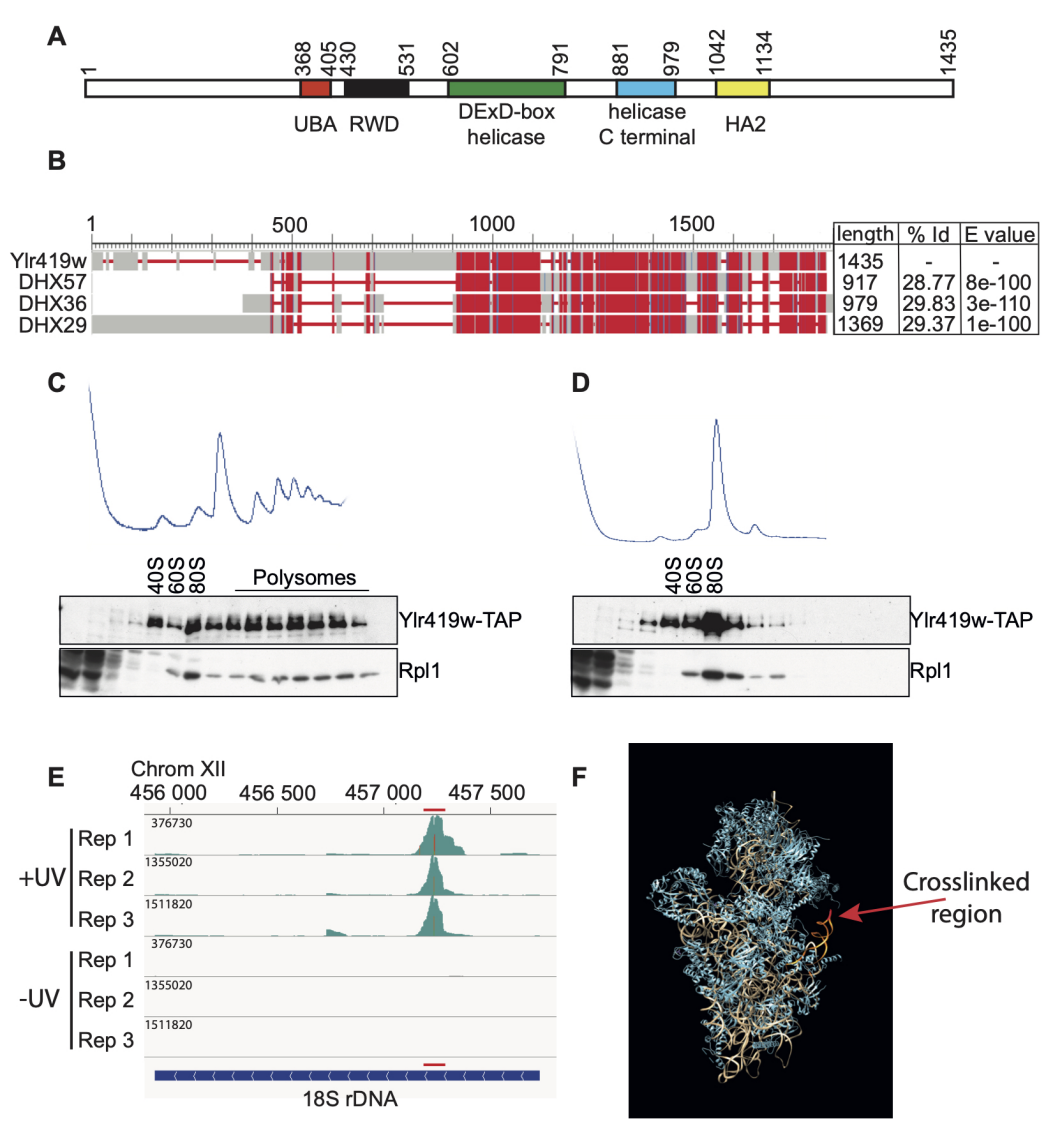
**A. Identification of protein domains.** Protein domains were identified using the SMART online tool (http://smart.embl.de/). All of them have a E-value < 2. 10 ^-5^ . **B. Alignment of the three best human homologues of Ylr419w.** It was done with BLAST software. The length of each protein, the percentage of identity, and the E value are indicated, respectively on the right. **C. Ylr419w-TAP protein sediments in the 40S, 80S and polysomes fractions.** Cellular extracts were separated on a 10/50% sucrose gradient. Fractions of 0.5 ml were recovered, the proteins were precipitated with TCA and then resuspended in sample buffer. The proteins were separated on polyacrylamide gel, transferred on nitrocellulose membrane (Bio-Rad), and revealed using appropriate antibodies. **D. Ylr419w-TAP protein directly binds to the ribosome.** The cellular extracts were treated with RNase before loading on the sucrose gradient. The fractions were treated as in C. **E. Identification of Ylr419w binding site into the 18S rRNA sequence.** The histograms plotted using the Integrated Genome Viewer software (IGV 2.12.3), display the sequences identified on CRAC analysis in the 18S rDNA region. Replicates in condition +UV and -UV are shown. The maximum number of hits in the main peaks is mentioned and mutated position corresponding to crosslink points are colored (also visualized in red in F) **.** The red line defines Ylr419w binding region (also visualized in orange in F), which corresponds to the shortest sequence common to most reads of this peak. **F. Localization of Ylr419w binding sites in the three-dimensional structure of the 40S ribosomal subunit.** The Ylr419w binding sites identified by CRAC are shown in orange and the position of crosslinked points in red. The image was obtained with the UCSF Chimera program v1.17.3 (Pettersen et al. 2004) using the ribosomal structures described in Ben-Shem et al. (3U5D and 3U5E) (Ben-Shem et al. 2011).

## Description

*Saccharomyces cerevisiae* was the first eukaryote organism to be sequenced (Goffeau et al. 1996) . High-throughput sequencing of its nuclear genome revealed that this yeast contains 6579 ORFs. Some of them are still dubious, but 5904 have been verified. Of these, several code proteins of still unknown function. 5242 have a gene name that assigns the product of the gene to a function, a pathway or a complex, but 663 are of completely unknown function ( https://yeastmine.yeastgenome.org/yeastmine/begin.do ).

YLR419W is an ORF coding a protein of 1435 AA with a molecular weight of 163 kDa. To discover the function of this protein, we first looked at the primary sequence and we searched for potential interacting domains or domains characteristic of a particular function using SMART tool (Letunic et al. 2021) **( Figure 1A )** . We observed that Ylr419wp contains a ubiquitin-associated domain (UBA) from position 368 to 405. This domain binds ubiquitin or polyubiquitin chains, which assist target proteins for degradation by the proteasome (Buchberger 2002 for review) **.** Near the UBA domain, the region from the position 430 to 531 contains an RWD domain that is found in a number of helicase (Doerks et al. 2002) . The central region of the protein from 602 to 791 contains a helicase ATP-binding DEAD-like domain. The region from 881 to 979 contains a helicase C terminal domain and the region from 1042 to 1134 contains a helicase associated domain (HA2). All these domains were identified with a E-value < 2 10 ^-5^ and are related to the superfamily 2 DEAH/DExH-box helicases, which mainly bind to RNA duplexes to participate in the ATP-dependent unwinding of these RNA molecules (Valentini and Linder 2021 and references therein). Secondly, we used BLAST to determine whether this protein could have a human homologue (https://blast.ncbi.nlm.nih.gov). The results of this search proposed three potential homologs, DHX29, DHX36 and DHX57 **( Figure 1B )** . DHX29 is involved in the unwinding of the structured 5’UTR of mRNAs during the 40S subunit scanning and contributes to the translation initiation pathway (Pisareva et al. 2008; Parsyan et al. 2009) . DHX36 binds to RNA and DNA G-quadruplex (G4) and is involved in many cellular processes including transcription, genome stability and translation (Schult and Paeschke 2021 for review; Antcliff et al. 2021 for review) **.** The function of the putative RNA helicase DHX57 is unknown. It has been observed that its expression level is increased in senescent cells (Piletska et al. 2022) **.** To discriminate between these three potential candidates, we first performed a sucrose gradient with polysome extracts from a strain expressing a C-terminal Tandem-Affinity-Purification (TAP) fusion of Ylr419w ( **Figure 1C** ). We observed that the Ylr419w-TAP fusion protein sedimented mainly in the 80S peak, but also in the polysome fractions and in the 40S fraction. This sedimentation profile suggested that Ylr419wp might be associated with the ribosome and preferentially with the 40S subunit ( **Figure 1C** ). We repeated the experiment using cell extracts that had previously been treated with RNAse. The RNAse treatment destroyed the polysomes and generated an important 80S peak. We observed that Ylr419w-TAP was accumulated in the 80S and 40S peaks but was not present in the free fractions of the gradient suggesting that Ylr419wp could be directly associated with the ribosome ( **Figure 1D** ). Therefore, these preliminary results pointed to DHX29 as the best putative Ylr419wp homolog. However, the two others candidates could not be exclude because the function of DHX57 is unknown and it was reported that DHX36 was also involved in the translation initiation of some mRNAs containing rG4 in their 5’-untranslated region (UTR) (Chen et al. 2021) . To gain further information, we performed a crosslinking and analysis of cDNA (CRAC) experiment using a Ylr419w-His6-TEV-Protein A (Ylr419w-HTP) strain (adapted from Granneman et al. 2009). It requires a direct interaction between Ylr419w-HTP and the RNA, as the bound RNAs are covalently attached to Ylr419w-HTP by UV crosslink, immunoprecipitated, moderately digested and finally identified by deep sequencing. This technique applied to Ylr419wp allowed the characterization of the contact sites, identified by a clear punctual deletion/mutation at the locus of contact, corresponding to the mark of the crosslink point after proteinase K digest. This defines the precise regions in contact between Ylr419w-HTP and the RNAs. We identified the strongest signal encompassing a precise region in the 18S ribosomal RNA from position 467 to 519 in the 18S rRNA (orange), with crosslink point at position 492 to 494 (red, **Figure 1E** ). This signal was not present in the control corresponding to the same sample without UV treatment.

Thanks to the structure of the ribosome, we localized the 18S region associated with Ylr419wp. This region, indicated by an arrow ( **Figure 1F** ), corresponds to the H16 helix located outside the protein-rRNA condensate block. Comparison of this position with the cryo-EM localization of DHX29 on the 40S subunit showed that both proteins bind precisely to the same region (Sweeney et al. 2021) **.**

These results showed that Ylr419wp is the yeast structural homolog of mammalian DHX29 translation initiation factor. It would be of interest to determine whether the translation of mRNA reporter containing strong structured 5’-UTR could be affected by the absence of Ylr419wp.

## Methods

Yeast strains

Ylr419w-TAP strain LMA2653 ( *MATa, ura3∆0, his3∆1, leu2∆0, met15∆0, YLR419W-TAP:HIS3MX6* ) used for the polysome gradient was obtained from the *S. cerevisiae* fusion library (Ghaemmaghami et al. 2003) .

The Ylr419w-HTP strain LMA3329 used for the CRAC experiment was obtained by transformation of the Ylr419w-TAP strain with the HpaI linearized plasmid pGIM1287.

This transformation resulted in the replacement of the *TAP:HIS3MX6* cassette by the *HTP:URA3MX6* cassette.

The Ucp12-HTP strain LMA4479 strain used for the *S. pombe* spike-in the CRAC experiment was obtained by transformation of a wild-type *S. pombe* strain by a GB1238-GB1239 PCR product from a NAT-HTP plasmid pGIM1359 with 50 bp flanking arms at the SPCC895.09c locus ( *Ucp12* ).

Plasmids

Plasmid pGIM1287 TAP2CRAC was obtained by the ligation of a 2191 pb purified CS883-CS884 PCR product on pBS1539-HTP (Granneman et al. 2009) with a Stu1 linearized PCR Blunt plasmid using Quick ligase (NEBiolabs) according to the manufacturer recommendations.

Plasmid pGIM1359 HTP-NAT for CRAC in *S. pombe* was obtained by the ligation into pBS1539 of an Apa1/HindIII digestion of a GB1125-GB1126 PCR product on a nourseothricin resistance [ *NAT ^R^* ] cassette, contained on the plasmid pAG25 (Goldstein and McCusker 1999) .

Polysomes gradient

Ylr419w-TAP strain was cultivated in 200 ml of rich medium (YPD) until OD _600_ 0,6. After treatment with 50 µg/ml cycloheximide for 5 min. at 4°C to block translation elongation, the cells were recovered by centrifugation at 4°C. They were resuspended in breaking buffer (20 mM Tris-HCl, pH 7.4, 50 mM KCl, 5 mM MgCl2, 50 µg/ml cycloheximide, and EDTA-free protease inhibitors from Roche) and broken with glass beads using in MagNA lyser (3 times at 3000 rpm for 1 min.). The lysate was clarified by centrifugation and 10 A _260_ were loaded on sucrose gradient (10/50%). To eliminate the polysomes, 10 A _260_ of cellular extracts were treated with 2µl of RNace It cocktail (Agilent) before loading on sucrose gradient. After centrifugation for 2h45 min. at 190,000g (SW41 Beckman Coulter), the fractions were recovered, and proteins were precipitated with 10% TCA. The proteins were resuspended in 40 µl of sample buffer and 10 µl were separated on polyacrylamide gel (NuPAGE 4-12% Bis-Tris Gel from Invitrogen), transferred on nitrocellulose membrane (Bio-Rad) and Ylr419w-TAP and Rpl1 proteins were detected using PAP at 1/5,000 dilution (Sigma-Aldrich) and anti Rpl1 (a gift from François Lacroute) antibodies respectively. The proteins were revealed with the clarity ECL substrate (Bio-Rad).

CRAC experiment

In vivo CRAC experiments were performed in triplicate as described previously (Granneman et al., 2009) with minor modifications described hereafter : 1) we added a spike of 10% Ucp12-HTP *S. pombe* cells carrying an HTP fusion to *YLR419W* homologs in *S. pombe* , 2) all the steps from 3’ primer ligation to 5’ linked ligation were performed directly on sepharose beads and radiolabelling was omitted, 3) SDS-Page purification was replaced by a GelFree separation device (Expedeon) using 5% Tris acetate cartridges. The fractions corresponding to Ylr419w-HTP molecular weight window were collected between 42 and 101 min of run at 100V. Primer used for 5’ ligation are respectively GB1043 GB1046 and GB1048 for +UV replicate 1, 2 and 3, and GB1044 GB1045 and GB1047 for -UV replicate 1, 2 and 3.

Library were amplified by PCR using GX1 and GX2 primers, and a customed number of cycles of amplification (19 to 23 cycles). Sequencing was performed on a Hiseq2500 (Illumina).

CRAC Data analysis

The data analysis was done as described in (Zhang et al. 2019 and references therein). Briefly, the quality of sequence data was visualized with FastQC v0.11.3 ( http://www.bioinformatics.babraham.ac.uk/projects/fastqc/ ). Single-end read duplicates were discarded using fqduplicate ( ftp://ftp.pasteur.fr/pub/gensoft/projects/fqtools ). Adaptor clipping and quality trimming of reads were performed using AlienTrimmer v0.4. Demultiplexing fastq files was done using Biopython v1.69. Trimmed reads were aligned along the *S. cerevisiae* S288C genome and the *S. pombe* genome using single-end mode of Bowtie2 using local read alignment with option ‘--sensitive-local’. Output SAM files were converted to BAM files using SAMtools v0.1.19. Data visualization was done with the Integrative Genomics Viewer (IGV) software.

**Data availability**

The genomic CRAC data were deposited on the GEO server under the accession number GSE252043.

## Reagents

*Table of oligonucleotides used in this study*

**Table:** 

CS883	AGTTAACCGCTTTAAGAAAATCTCATCCTCCGGGGCACTTGATTATGATggatccatggagcaccatca
CS884	TGTTAACTGGATGGCGGCGTTAGTATCGAATCGACAGCAGTATAGCGACCAGCAatacgactcactatagggcg
GX1	CAAGCAGAAGACGGCATACGA
GX2	AATGATACGGCGACCACCGACAGGTTCAGAGTTCTACAGTCCGACGA
GB1043	5InvddT/GTTCArGrArGrUrUrCrUrArCrArGrUrCrCrGrArCrGrArUrCrNrNrNrGrArUrN
GB1044	5InvddT/GTTCArGrArGrUrUrCrUrArCrArGrUrCrCrGrArCrGrArUrCrNrNrNrCrUrGrN
GB1045	5InvddT/GTTCArGrArGrUrUrCrUrArCrArGrUrCrCrGrArCrGrArUrCrNrNrNrCrGrUrN
GB1046	5InvddT/GTTCArGrArGrUrUrCrUrArCrArGrUrCrCrGrArCrGrArUrCrNrNrNrGrCrArN
GB1047	5InvddT/GTTCArGrArGrUrUrCrUrArCrArGrUrCrCrGrArCrGrArUrCrNrNrNrTrGrArN
GB1048	5InvddT/GTTCArGrArGrUrUrCrUrArCrArGrUrCrCrGrArCrGrArUrCrNrNrNrArCrGrN
GB1125	ggaagtcaacctgaagcttgataATAGGCCACTAGTGGATCTG
GB1126	gggtaccgggcccggagacaatcCGGATCCCCGGGTTAATTAA
GB1238	TTAAACTACGAAAGTGAAATACATCAATGTATTCGTACTCTGATTGCAGGCAACGGTGTTtccatggagcaccatca
GB1239	gaacaataaaagtgatttgtaccatttatgctctttcaaggaaatagagaaggggagCTAtacgactcactatagggcgaa
